# My Calling

**DOI:** 10.1007/s11606-025-09618-z

**Published:** 2025-06-30

**Authors:** William M. Tierney

**Affiliations:** 1 Richard M. Fairbanks School of Public Health, Indiana University Indianapolis, Indianapolis, IN USA; 2 Regenstrief Institute, Inc., Indianapolis, IN USA; 3 Indianapolis, USA

I

am

to be

the one

who is there

when pain, fear,

and angst prevail and

overwhelm and frighten the

sick, the desperate, and those whose

needs must be first, must be met, but met

with inadequate time, space, knowledge, love,

and compassion that can only come from a calling to

serve, to be there, to hold hands and comfort eyes that

have seen too much, understood too little, with a dearth

of patience, caring, wanting to be the one in the breach,

holding back the dark, pain, and a lack of trust in those

who have promised much, delivered too little to ease

the miseries of life that they share and we can only

try to understand through caring for and about

them during difficult, fearful times, sitting

anxiously in our waiting rooms,

waiting for the doctor.

